# Efficacy and Safety of Cold Atmospheric Pressure Plasma in Dental Practice: A Systematic Review of Preclinical and Clinical Evidence

**DOI:** 10.1155/ijod/7100799

**Published:** 2026-05-30

**Authors:** Shreya Hegde, Vijaya Hegde, Pooja J. Shetty, Chitharanjan M. Shetty, Sandya Kini, Neetha J. Shetty, Ayan B. Ray

**Affiliations:** ^1^ Department of Conservative Dentistry and Endodontics, Manipal College of Dental Sciences Mangalore, Manipal Academy of Higher Education, Manipal, India, manipal.edu; ^2^ Department of Public Health Dentistry, A. J. Institute of Dental Sciences, NH-66 Near Kuntikana Road, Kuntikana, Mangalore, 575004, Karnataka, India, ajdental.in; ^3^ Department of Conservative Dentistry and Endodontics, A. B. Shetty Memorial Institute of Dental Sciences, Nitte Deemed to be University, Deralakatte, Mangalore, 575018, Karnataka, India, nitte.edu.in; ^4^ Department of Conservative Dentistry and Endodontics, Manipal College of Dental Sciences, Manipal Academy of Higher Education, Manipal, India, manipal.edu; ^5^ Department of Periodontics, Manipal College of Dental Sciences Mangalore, Manipal Academy of Higher Education, Manipal, India, manipal.edu; ^6^ Consultant Endodontist, Kolkata, West Bengal, India

**Keywords:** antimicrobial therapy, bonding strength, cold atmospheric pressure plasma, dentistry, periodontitis, regeneration, safety

## Abstract

**Introduction:**

Cold atmospheric pressure plasma (CAPP) has emerged as a promising nonthermal technology for antimicrobial disinfection and tissue modulation in dentistry. Its reactive oxygen and nitrogen species (RONS) facilitate the destruction of microorganisms, the disruption of biofilms, and the biomodulation of cells and tissues without causing thermal harm. The lack of translation into clinical practice despite the large number of in vitro investigations calls for a thorough synthesis of the available data.

**Objective:**

To systematically evaluate the efficacy and safety of CAPP across dental applications based on preclinical (in vitro and animal) and clinical studies.

**Materials and Methods:**

This review was prospectively registered in the International Prospective Register of Systematic Reviews (PROSPERO) (CRD420251154333) and adhered to Preferred Reporting Items for Systematic Reviews and Meta‐Analyses (PRISMA) guidelines. A comprehensive search was conducted in PubMed, MEDLINE, Scopus, Cochrane CENTRAL, and Web of Science. Inclusion criteria encompassed in vitro, ex vivo, animal, and human studies employing CAPP or plasma‐activated liquids (PALs) for dental applications. Outcomes included antimicrobial efficacy, remineralization, bond strength, wound healing, and safety.

**Results:**

Nineteen studies met the inclusion criteria. Consistent antibacterial, remineralizing, and bonding‐enhancing effects were shown in in vitro tests, along with significant increases in fibroblast proliferation and wound healing capability. Long‐term exposure safety and a decrease in alveolar bone loss, peri‐implant bacterial load, and periodontal inflammation were validated by in vivo investigations. No cytotoxic or carcinogenic effects were reported.

**Conclusions:**

Evidence from included studies, which were predominantly preclinical, suggests that CAPP demonstrates antimicrobial, bonding, remineralization, and regenerative potential with a favorable safety profile. However, clinical evidence is limited to a single short‐term randomized trial. Therefore, conclusions regarding routine clinical application remain preliminary, and further well‐designed clinical studies are required.

## 1. Introduction

Plasma, often referred to as the fourth state of matter, is a partially or fully ionized quasi‐neutral medium composed of electrons, ions, neutral particles, radicals, and photons that collectively exhibit complex behavior. It constitutes more than 99% of the universe’s observable matter. Plasmas are categorized as thermal (equilibrium/hot) or nonthermal (nonequilibrium/cold) based on the temperature equilibrium between electrons, ions, and neutrals. Weakly ionized gases produced at atmospheric or low pressures are known as cold plasmas [1]. Cold atmospheric pressure plasma (CAPP) is a new nonthermal technology that produces a mixture of reactive oxygen and nitrogen species (RONS), electrons, ions, and charged particles at temperatures close to room temperature. Without causing thermal damage to biological tissues, these reactive species have strong antibacterial, biomodulatory, and surface‐modifying properties [2–4]. Through regulated plasma–tissue interaction, CAPP allows for contactless disinfection and biological stimulation, in contrast to traditional chemical disinfectants or thermal modalities. CAPP, which was first created for sterilization and biomedical surface applications, has drawn more attention in plasma medicine because of its capacity to control redox homeostasis, encourage wound healing, and cause selective cytotoxicity against cancerous and infectious cells [3, 4].

The clinical adaptability of atmospheric plasma systems, including dielectric barrier discharges (DBDs) and atmospheric pressure plasma jets (APPJ), has been investigated in dentistry. They have been utilized to speed up mucosal healing, improve adhesive bonding to enamel and dentin, encourage periodontal regeneration, and clean infected root canals. CAPP efficiently decreases bacterial and fungal biofilms, such as *Streptococcus mutans*, *Enterococcus faecalis*, and *Candida albicans*, while encouraging fibroblast migration, type I collagen synthesis, and angiogenic signaling [5, 6]. Furthermore, plasma treatment increases surface wettability and energy of dental substrates, thereby improving resin adhesion. Its nonthermal antimicrobial properties also support its use in minimally invasive caries management and endodontic disinfection. Animal and early clinical studies have reported improved periodontal outcomes, reduced microbial recolonization, and favorable wound healing responses without significant cytotoxic or mutagenic effects [5–7]. Despite these promising findings, the body of evidence is still dispersed. Establishing standard therapy parameters and comparing results are challenging as the majority of the studies are small‐scale laboratory trials with varied plasma sources, gases (He, Ar, N_2_, or air), exposure durations, and treatment regimens [7, 8]. Moreover, while CAPP shows a high degree of biological selectivity, the long‐term safety and reproducibility of its clinical applications are yet to be validated. A rigorous synthesis of in vitro, in vivo, and early clinical findings is necessary to elucidate the translational potential of the fast‐growing yet diverse literature. The objective of this review is to systematically assess CAPP’s safety and effectiveness in dental applications based on preclinical (in vitro and animal) and clinical investigations.

## 2. Materials and Methods

### 2.1. Protocol and Registration

This systematic review was conducted following the Preferred Reporting Items for Systematic Reviews and Meta‐Analyses (PRISMA) 2020 statement [9]. The protocol was prospectively registered with the International Prospective Register of Systematic Reviews (PROSPERO) under Registration Number CRD420251154333.

### 2.2. Eligibility Criteria

Eligibility criteria were framed using the PICOS framework. This review included in vitro, animal, and human studies to enable a comprehensive evaluation of mechanistic, preclinical, and early clinical evidence. The PICOS framework was adapted to accommodate preclinical experimental models, given the emerging nature of this field.

#### 2.2.1. Population

Clinical models including dental tissues, oral mucosa, or biofilms in humans, animals, and laboratories (in vitro and in vivo).

#### 2.2.2. Intervention

The use of plasma‐activated liquids (PALs) or CAPP in dental or oral applications for antibacterial, bonding, remineralization, or wound healing purposes.

#### 2.2.3. Comparator

Traditional methods (e.g., disinfectants, fluoride varnish, scaling and root planing, or untreated controls).

#### 2.2.4. Outcomes

##### 2.2.4.1. Primary Outcomes

Antimicrobial activity, enamel/dentin remineralization, bonding enhancement, tissue regeneration, and safety.

##### 2.2.4.2. Secondary Outcomes

Cellular and histological changes, biochemical markers, and morphological effects.

#### 2.2.5. Study Designs

Original, peer‐reviewed research conducted on humans, animals, in vitro, and ex vivo.

#### 2.2.6. Exclusion Criteria

Review articles, conference abstracts, editorials, simulations, and studies not specifically centered on dental or oral plasma uses were excluded.

### 2.3. Information Sources and Search Strategy

A systematic search was performed across PubMed/MEDLINE, Scopus, Cochrane CENTRAL, and Web of Science from January 2015 to September 2025. Searches were limited to English‐language articles. The electronic search strategy was developed using a combination of controlled vocabulary (MeSH terms) and free‐text keywords to maximize sensitivity and comprehensiveness. The PubMed search strategy was first constructed and subsequently adapted for Scopus, Cochrane CENTRAL, and Web of Science using database‐specific syntax. The PubMed search strategy was as follows: (“Plasma Gases”[Mesh] OR “cold atmospheric plasma” OR “cold atmospheric pressure plasma” OR “non‐thermal plasma” OR “nonthermal plasma” OR “plasma medicine” OR “plasma therapy” OR “plasma jet” OR “atmospheric pressure plasma” OR “dielectric barrier discharge” OR “plasma‐activated liquid” OR “plasma‐activated water”) AND (“Dentistry”[Mesh] OR “Mouth”[Mesh] OR “Oral Health”[Mesh] OR “Tooth”[Mesh] OR dentistry OR dental OR oral OR tooth OR teeth OR enamel OR dentin OR biofilm OR “dental biofilm” OR periodontitis OR periodontal OR implant OR “peri‐implantitis” OR mucosa OR “oral mucosa”). Boolean operators (AND, OR) were used to combine concepts, and truncation and phrase searching were applied where appropriate. Synonyms and variations of key terms (e.g., “nonthermal plasma” vs. “non‐thermal plasma”) were included to ensure comprehensive retrieval. In Scopus, the TITLE‐ABS‐KEY fields were used to capture relevant records using equivalent keywords and Boolean operators. In Web of Science, a topic search (TS = ) was employed. For Cochrane CENTRAL, a simplified combination of keywords was applied, with filters used to identify relevant clinical and experimental studies. In addition, manual searching of reference lists of included studies and relevant reviews was conducted to identify any additional eligible articles not captured through database searches. Details of the search strategy are shown in Table 1.

**Table 1:** Search strategy and retrieval summary.

Source	Search period	Search string	Filters applied	Records retrieved (*n*)
PubMed/MEDLINE	January 2015–September 2025	(“Plasma Gases”[Mesh] OR “cold atmospheric plasma”[tiab] OR “cold atmospheric pressure plasma”[tiab] OR “non‐thermal plasma”[tiab] OR “nonthermal plasma”[tiab] OR “plasma medicine”[tiab] OR “plasma therapy”[tiab] OR “plasma jet”[tiab] OR “atmospheric pressure plasma”[tiab] OR “dielectric barrier discharge”[tiab] OR “plasma‐activated liquid”[tiab] OR “plasma‐activated water”[tiab]) AND (“Dentistry”[Mesh] OR “Mouth”[Mesh] OR “Oral Health”[Mesh] OR “Tooth”[Mesh] OR dentistry[tiab] OR dental[tiab] OR oral[tiab] OR tooth[tiab] OR teeth[tiab] OR enamel[tiab] OR dentin[tiab] OR biofilm[tiab] OR “dental biofilm”[tiab] OR periodontitis[tiab] OR periodontal[tiab] OR implant[tiab] OR implants[tiab] OR “peri‐implantitis”[tiab] OR mucosa[tiab] OR “oral mucosa”[tiab])	English; original studies	102
Scopus	January 2015–September 2025	TITLE‐ABS‐KEY (“cold atmospheric plasma” OR “cold atmospheric pressure plasma” OR “non‐thermal plasma” OR “nonthermal plasma” OR “plasma medicine” OR “plasma therapy” OR “plasma jet” OR “atmospheric pressure plasma” OR “dielectric barrier discharge” OR “plasma‐activated liquid” OR “plasma‐activated water”) AND TITLE‐ABS‐KEY (dentistry OR dental OR oral OR tooth OR teeth OR enamel OR dentin OR biofilm OR “dental biofilm” OR periodontitis OR periodontal OR implant OR implants OR “peri‐implantitis” OR mucosa OR “oral mucosa”)	English; research articles	58
Cochrane CENTRAL	January 2015–September 2025	(“cold atmospheric plasma” OR “cold atmospheric pressure plasma” OR “non‐thermal plasma” OR “plasma jet” OR “plasma therapy”) AND (oral OR dental OR dentistry OR tooth OR enamel OR dentin OR biofilm OR periodontitis OR implant OR mucosa)	Trials only; English	21
Web of Science	January 2015–September 2025	TS = (“cold atmospheric plasma” OR “cold atmospheric pressure plasma” OR “non‐thermal plasma” OR “plasma jet” OR “atmospheric pressure plasma” OR “dielectric barrier discharge” OR “plasma‐activated liquid”) AND TS = (dent ^∗^ OR oral OR tooth OR enamel OR dentin OR biofilm OR periodontitis OR implant ^∗^ OR mucosa)	English; articles	33
Citation Searching	January 2015–September 2025	Manual screening of reference lists of all included studies and relevant review articles to identify additional eligible records not retrieved through database searches	Not applicable	10
Total studies retrieved				214

### 2.4. Study Selection and Data Extraction

Data screening and extraction were conducted using the Covidence software application [10]. Two stages of screening were conducted: Title and abstract screening, followed by a full‐text evaluation of potentially eligible articles. Based on the predetermined criteria, eligibility was evaluated independently by two reviewers. Any disagreements were resolved by discussion or, if necessary, adjudication by a third reviewer. Extracted information included study details (author, year), study design and experimental model, type of plasma device (DBD, APPJ, or modified system), carrier gas type (He, Ar, N_2_, or air), comparator group, outcome measures, and key results.

### 2.5. Risk of Bias (RoB) Assessment

Two reviewers independently evaluated the RoB using validated instruments tailored to the study design: A modified CONSORT checklist was used to evaluate in vitro research, evaluating randomization, control inclusion, replicates, blinding, completeness of outcome reporting, and statistical analysis [11, 12]. SYRCLE’s RoB tool was used to assess animal studies evaluating sequence generation, allocation concealment, baseline similarity, blinding, incomplete data, and selective outcome reporting [13]. Clinical studies were assessed using the Cochrane RoB 2.0 tool [14], which looked at randomization process, deviations from intended interventions, missing data, measurement of outcomes, and selective reporting. Every domain received a rating of either high risk, unclear risk, or low risk. All differences were discussed, and a consensus was reached.

### 2.6. Data Synthesis

Meta‐analysis was not possible because of the heterogeneity in plasma devices, gases, exposure durations, and results. A narrative synthesis approach was adopted. Both in vitro and in vivo studies were included. The findings were categorized thematically under five domains: antimicrobial/biofilm control, remineralization/hard tissue effects, bonding and adhesion enhancement, soft‐tissue healing/regeneration, and safety/biocompatibility.

## 3. Results

### 3.1. Study Selection

**Figure 1:**
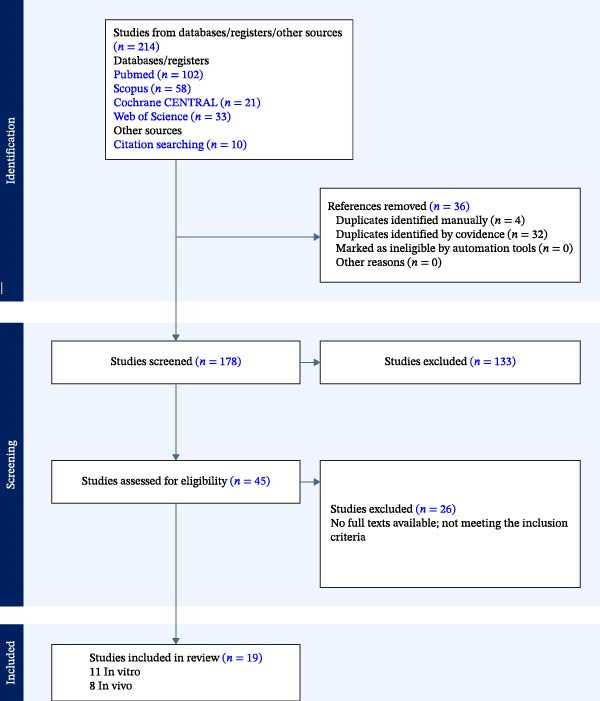
PRISMA 2020 flow diagram.

The initial database search retrieved 214 records. Following the elimination of duplicates (*n* = 36), 178 distinct articles were filtered based on abstract and title. After 45 full‐texts were evaluated for eligibility, 19 studies were included: 11 in vitro studies, 7 animal studies, and 1 human randomized clinical trial. To facilitate interpretation, findings are presented separately as preclinical (in vitro and animal) and clinical evidence. Figure 1 summarizes the PRISMA information flow via the review process. The CAPP devices that were employed in various studies were APPJ and DBD systems, which mainly used air, argon, or helium as carrier gases. Durations of exposure varied from 10 s to 5 min, contingent on the model and treatment goal. As outlined in Tables 2–4, the results were divided into five domains: antibacterial activity, bonding and adhesion, remineralization, soft‐tissue regeneration, and safety/biocompatibility.

**Table 2:** Summary table of study characteristics of in vitro studies (*n* = 11).

Author (year)	Model/type	Intervention/plasma type	Outcome measures	Key findings
Silva et al. (2024) [15]	Human gingival fibroblasts	Helium‐based CAPP activation	Cell proliferation, migration, wound healing	Increased fibroblast proliferation and migration
Shafigh et al. (2025) [16]	Human enamel	Plasma pretreatment before ceramic/composite bonding	Shear bond strength	Improved enamel–resin bond strength
Bakhtiary et al. (2025) [17]	Enamel blocks	Nanohydroxyapatite ± low‐power laser or CAPP	Microhardness, SEM	Enhanced remineralization and surface hardness
Suhail Ali et al. (2024) [18]	*S. mutans* isolates	Air‐based CAP jet exposure	CFU reduction, SEM	Dose‐dependent bacterial reduction
Kermanshah et al. (2020) [19]	Dentin discs	Helium plasma jet ± cavity liners	Microhardness, remineralization	Increased dentin remineralization (with liners)
Abdul Salam Ibrahim et al. (2023) [20]	Mixed oral bacteria	Plasma jet (argon)	CFU, inhibition zone	Broad‐spectrum antimicrobial effect
Kumar et al. (2023) [21]	*E. faecalis*‐infected root canals	CAP jet disinfection	Microbial counts, SEM	Effective root canal disinfection
Lim et al. (2025) [22]	*S. mutans* biofilm	Air CAP exposure	Biofilm viability, CFU	Significant biofilm inactivation
Khoubrouypak et al. (2021) [23]	Human enamel	CAPP + resin/xylitol varnish	Hardness, erosion resistance	Reduced enamel erosion
Qi et al. (2022) [24]	Caries‐affected dentin	Helium CAP jet	Bond strength, XPS, wettability	Improved bonding and surface wettability
Görgen et al. (2023) [25]	Abutment–crown interface	CAP + surface pretreatments	Pull‐off tensile load	Increased tensile bond strength

**Table 3:** Summary of study characteristics of in vivo studies (*n* = 8).

Author (year)	Model/population	Intervention/plasma source	Outcome measures	Key findings
Borges et al., 2018 [26]	Mice–oral candidiasis	Helium amplitude‐modulated CAP jet	CFU, histology, inflammation	Reduced fungal load and inflammation
Lima et al., 2021 [28]	Rat–periodontitis	Helium CAP jet adjunctive to therapy	CAL, bone loss, microbial count	Improved periodontal healing and reduced bacterial load
Kusakcı‐Seker et al., 2021 [29]	Rat–experimental periodontitis	Nonthermal plasma (gas NR)	Bone loss, oxidative stress	Decreased bone loss with antioxidant effect
Evert et al., 2021 [32]	Mouse–safety study	Cold plasma exposure for 12 months	Histopathology	No carcinogenic or dysplastic changes
Zhang et al., 2018 [27]	Rat–ligature‐induced periodontitis	Nonthermal plasma	Bone morphology, histology	Reduced bone loss and inflammation
Shi et al., 2015 [30]	Rat–peri‐implantitis	Nonequilibrium plasma jet	Bone‐to‐implant contact	Enhanced bone regeneration
Zhou et al., 2022 [31]	Beagle dogs–peri‐implantitis	Modified CAPP (MCAP)	Bone volume, histology, bacterial load	Reduced bone loss and bacterial load
Küçük et al., 2020 [33]	Humans–chronic periodontitis	Nonthermal plasma adjunct to SRP	PD, CAL, PI, GI	Improved clinical periodontal parameters

**Table 4:** Summary of studies by application domain of cold atmospheric pressure plasma (CAPP).

Domain	Number of Studies	Studies	Key findings
Antimicrobial/biofilm control	7	Suhail Ali et al., [18]; Kumar et al., [21]; Abdul Salam Ibrahim et al., [20]; Lim et al., [22]; Borges et al., [26]; Zhang et al., [27]; Lima et al., [28]	Consistent reduction in microbial load and biofilms
Bonding/adhesion enhancement	3	Shafigh et al., [16]; Qi et al., [24]; Görgen et al., [25]	Increased bond strength and surface wettability
Remineralization/hard tissue effects	3	Bakhtiary et al., [17]; Kermanshah et al., [19]; Khoubrouypak et al., [23]	Improved mineralization and surface hardness
Wound healing/cell proliferation	1	Silva et al., [15]	Enhanced fibroblast activity and wound healing
Periodontal/peri‐implant regeneration	4	Lima et al., [28]; Kusakcı‐Seker et al., [29]; Shi et al., [30]; Zhou et al., [31]	Reduced inflammation and bone loss
Safety/biocompatibility	1	Evert et al., 2021 [32]	No adverse histological effects

### 3.2. Preclinical Evidence (In Vitro and Animal Studies)

#### 3.2.1. Evidence From In Vitro Studies

A total of 11 in vitro [15–25] evaluated CAPP across multiple dental applications, including antimicrobial efficacy, surface modification, and cell‐level bioactivity.

##### 3.2.1.1. Antimicrobial and Biofilm Effects

The ability of CAPP to combat oral infections such as *S. mutans*, *E. faecalis*, and *C. albicans* was examined in seven different trials. Significant biofilm reduction and microbial inactivation were shown by Suhail Ali et al. [18] and Lim et al. [22] after exposure to helium‐ or air‐based plasma jets; the efficacy of these treatments was directly correlated with the duration of exposure. CAPP’s promise as an adjuvant endodontic disinfectant was supported by a study by Kumar et al. [21], which showed that it significantly disinfected root canals infected with *E. faecalis*. Abdul Salam Ibrahim et al. [20] observed broad‐spectrum inhibition of cariogenic bacteria, with no residual toxicity. Collectively, these results point to CAPP’s potent, repeatable antimicrobial activity against a variety of microbial species and substrates, which is caused by RONS that break down bacterial membranes and biofilm matrices.

##### 3.2.1.2. Bonding and Surface Modification

The function of plasma in improving the adherence of restorative materials was investigated in three studies: Shafigh et al. [16] demonstrated that the shear bond strength of ceramic and composite resins was considerably enhanced by plasma pretreating enamel surfaces. According to Qi et al. [24], helium CAP increased surface wettability and eliminated smear layers, which facilitated bonding to simulated caries‐affected dentin. When CAPP was used in conjunction with traditional surface treatments, Görgen et al. [25] showed an increase in the tensile bond strength between crowns and abutments. Mechanistically, these improvements were attributed to increased surface energy, microcleaning effects, and the formation of active functional groups facilitating better resin infiltration.

##### 3.2.1.3. Remineralization and Hard‐Tissue Effects

Three studies [17, 19, 23] assessed the impact of plasma on the remineralization of enamel and dentin. While CAPP by itself improved surface mineral deposition and microhardness, combination treatments with fluoride varnish or nanohydroxyapatite produced synergistic effects that strengthened enamel’s resistance to demineralization and erosion. CAPP is positioned as a promising noninvasive adjunct for restorative and preventive dentistry based on these findings.

##### 3.2.1.4. Cellular Response and Biocompatibility

The biological reaction of human gingival fibroblasts exposed to cold plasma was examined by Silva et al. [15] The study highlighted the biomodulatory and regenerative potential of plasma by showing a notable increase in cell migration, proliferation, and wound healing without cytotoxicity.

Overall, in vitro studies consistently confirmed that CAPP effectively reduces microbial load, enhances bonding, promotes remineralization, and supports cell viability. No significant adverse effects were reported at therapeutic doses.

#### 3.2.2. Evidence From In Vivo Studies

Seven animal studies [26–32] evaluated the therapeutic, regenerative, and safety aspects of CAPP.

##### 3.2.2.1. Periodontal and Peri‐Implant Applications

Multiple studies [27, 28, 30, 31] have consistently shown that the use of CAPP improved bone regeneration around periodontally impaired or peri‐implant locations, significantly reduced gingival inflammation and bacterial burden, and decreased alveolar bone loss. In treated tissues, histological examinations showed enhanced vascularization and thicker collagen bundles. Notably, modified CAPP (MCAP) improved bone volume fraction and decreased bacterial load in a beagle peri‐implantitis model, as demonstrated by Zhou et al [31].

##### 3.2.2.2. Anti‐Inflammatory and Regenerative Properties

Nonthermal plasma reduced oxidative stress and alveolar bone loss in experimental periodontitis, according to Kusakcı‐Seker et al. [29], while Lima et al. [28] saw synergistic gains in periodontal healing when plasma was administered as a supplement to traditional therapy.

##### 3.2.2.3. Antifungal and Mucosal Healing

Borges et al. [26] confirmed the antifungal properties of helium‐based plasma without causing mucosal toxicity by showing that it decreased *C. albicans* tissue invasion and inflammation in a mouse model of oral candidiasis.

##### 3.2.2.4. Long‐Term Clinical Effectiveness and Safety

Evert et al. [32] confirmed that repeated plasma exposure is safe over the long term by observing no signs of carcinogenicity or dysplastic change in the oral mucosa of mice after a year of therapy [33].

### 3.3. Clinical Evidence

A randomized clinical trial by Küçük et al. [33] showed that using plasma in addition to scaling and root planing significantly decreased probing depth, gingival inflammation, and microbial load, confirming its clinical applicability and safety in chronic periodontitis management.

### 3.4. Summary by Domain

Table 4 summarizes the key conclusions and evidence distribution across all 19 studies. Overall, the evidence generally points to CAPP’s multimodal therapeutic potential, which combines surface modification, tissue regeneration, and antibacterial activity, all while preserving a high level of safety in preclinical and clinical settings.

### 3.5. RoB

The RoB was evaluated using the modified CONSORT checklist for in vitro studies [11, 12], SYRCLE’s tool for animal studies [13], and Cochrane RoB 2 for the clinical trial [14]. The majority of in vitro investigations demonstrated low risk across domains, with three studies [16, 19, 25] having modest issues with blinding and randomization. All animal studies demonstrated low RoB, with adequate allocation, baseline similarity, and complete outcome reporting. The clinical trial [33] also showed low risk in all domains. Overall, the included studies exhibited good methodological quality, supporting the reliability of findings summarized in Tables 5 and 6.

**Table 5:** Risk of bias assessment for in vitro studies (modified CONSORT criteria).

Study	Randomization	Control use	Replicates	Blinding	Outcome reporting	Statistical methods	Overall risk
Silva et al., [15]	Low	Low	Low	Unclear	Low	Low	Low
Shafigh et al., [16]	Unclear	Low	Low	Unclear	Low	Low	Low
Bakhtiary et al., [17]	Unclear	Low	Low	Unclear	Low	Low	Low
Suhail Ali et al., [18]	Low	Low	Low	Unclear	Low	Low	Low
Kermanshah et al., [19]	Unclear	Low	Low	High	Low	Low	Moderate
Abdul Salam Ibrahim et al., [20]	Low	Low	Low	Unclear	Low	Low	Low
Kumar et al., [21]	Low	Low	Low	Unclear	Low	Low	Low
Lim et al., [22]	Low	Low	Low	Unclear	Low	Low	Low
Khoubrouypak et al., [23]	Low	Low	Low	Unclear	Low	Low	Low
Qi et al., [24]	Low	Low	Low	Unclear	Low	Low	Low
Görgen et al., [25]	Unclear	Low	Low	Unclear	Low	Low	Moderate

**Table 6:** Risk of bias assessment for in vivo studies (SYRCLE and RoB 2 criteria).

Study	Sequence generation	Baseline similarity	Allocation concealment	Blinding	Incomplete data	Selective reporting	Other bias	Tool used/overall risk
Borges et al., [26]	Low	Low	Low	Low	Low	Low	Low	SYRCLE—low
Lima et al., [28]	Low	Low	Low	Low	Low	Low	Low	SYRCLE—low
Kusakcı‐Seker et al., [29]	Low	Low	Low	Low	Low	Low	Low	SYRCLE—low
Evert et al., [32]	Low	Low	Low	Low	Low	Low	Low	SYRCLE—low
Zhang et al., [27]	Low	Low	Low	Low	Low	Low	Low	SYRCLE—low
Shi et al., [30]	Low	Low	Low	Low	Low	Low	Low	SYRCLE—low
Zhou et al., [31]	Low	Low	Low	Low	Low	Low	Low	SYRCLE—low
Küçük et al., [33]	Low	Low	—	Low	Low	Low	Low	RoB 2—low

### 3.6. Certainty of Evidence

Due to heterogeneity in study designs, interventions, and outcome measures, formal GRADE assessment was not feasible. However, a qualitative assessment indicated that in vitro studies provide high internal validity for mechanistic outcomes but lack clinical relevance, while animal studies offer moderate‐certainty evidence with limited generalizability. Clinical evidence remains low in certainty, as it is based on a single short‐term randomized trial. Overall, although preclinical findings support the biological effects and safety of CAPP, evidence for routine clinical use remains limited, warranting cautious interpretation and further clinical research.

### 3.7. Meta‐Analysis Feasibility

Outcome heterogeneity and variation in measurement tools precluded meta‐analysis.

## 4. Discussion

### 4.1. Principal Findings

This systematic review synthesizes evidence from 19 studies, the majority of which are in vitro and animal‐based, with only one randomized clinical trial. The findings indicate consistent biological and therapeutic effects of CAPP across experimental models; however, the predominance of preclinical evidence limits direct clinical extrapolation. The findings of this study align with earlier narrative reviews, such as those by Suresh et al. [6] and Borges et al. [5], which report the potential of CAPP for biofilm reduction, surface decontamination, and tissue effects without thermal damage.

### 4.2. Mechanisms of Action of CAPP

The biological effects of CAPP are attributed to its physicochemical composition, comprising charged particles, UV photons, electric fields, and RONS. These components are thought to interact with biological tissues and microorganisms, producing a range of effects; however, current mechanistic understanding is largely derived from in vitro and experimental models and should be interpreted with caution [5, 6]. RONS, including O_3_, H_2_O_2_, NO, and OH radicals, are considered central to the antimicrobial effects of CAPP, primarily through oxidative damage to microbial cellular components such as nucleic acids, proteins, and lipids. Experimental studies have demonstrated reductions in bacterial viability in *S. mutans*, *E. faecalis*, and *C. albicans*, including within biofilms [5], although clinical translation remains uncertain.

In addition to antimicrobial activity, plasma treatment has been shown in laboratory settings to modify surface wettability and energy by introducing polar functional groups on enamel, dentin, and restorative materials. This may facilitate improved resin infiltration and micromechanical retention, with in vitro studies reporting enhanced bond strength [16, 24]. Similarly, some evidence suggests that CAPP may contribute to remineralization by increasing surface microhardness and promoting mineral deposition, particularly when used in combination with fluoride or nanohydroxyapatite [17].

At the cellular level, CAPP has been reported to influence fibroblast migration and proliferation, potentially through modulation of redox‐sensitive pathways. In vitro findings, such as increased gingival fibroblast activity [15], suggest possible regenerative effects. Animal studies by Zhang et al. [27] and Lima et al. [28] further indicate that plasma exposure may be associated with reduced inflammatory markers and improved bone‐related outcomes in periodontal and peri‐implant models. However, these observations remain confined to preclinical settings and require validation in human studies.

### 4.3. Translational Insights and Clinical Implications

Although combined in vitro and in vivo findings suggest potential applicability of CAPP in dental practice, the current evidence base is predominantly preclinical. The single available randomized clinical trial [33] provides only preliminary support for its adjunctive use in periodontal therapy, and broader clinical implementation cannot be recommended at this stage. Future research should prioritize well‐designed, adequately powered randomized controlled trials to establish clinical efficacy, optimal treatment parameters, and long‐term safety.

Preclinical evidence indicates that CAPP may have relevance across multiple dental domains; however, these findings should be interpreted cautiously until supported by robust clinical data. In periodontics and implantology, experimental studies report reductions in inflammation, microbial load, and alveolar bone loss, while limited clinical evidence suggests short‐term improvements in periodontal parameters. In restorative dentistry, in vitro studies indicate enhanced surface characteristics and bonding performance [16, 24], though clinical validation is lacking. Similarly, antimicrobial and antibiofilm effects observed in endodontic models remain confined to laboratory settings. Evidence supporting roles in remineralization, mucosal healing, and regenerative processes is also derived primarily from preclinical studies [5, 15, 24].

The safety profile of CAPP appears favorable in experimental models, with no reported cytotoxic or carcinogenic effects under controlled conditions [32]. Limited human data suggest short‐term safety [33]; however, long‐term effects in clinical populations remain insufficiently investigated.

### 4.4. Methodological Strengths and Limitations

This review’s strengths include a robust synthesis, supported by a transparent protocol, dual‐reviewer screening, and comprehensive RoB assessment. The methodological quality of included studies was generally high. Most in vitro and animal studies demonstrated adequate controls, replication, and statistical reporting. This study has several limitations. First, the evidence base is heavily weighted toward in vitro and animal studies, with only one randomized clinical trial available, which limits the generalizability of findings to clinical practice. Second, substantial heterogeneity was observed across studies in terms of plasma devices, carrier gases, exposure durations, and outcome measures, precluding quantitative synthesis. Third, variability in experimental conditions limits comparability across studies and may influence observed effects. Finally, the possibility of publication bias cannot be excluded, as studies reporting positive outcomes may be more likely to be published.

### 4.5. Future Research Directions

Future research should prioritize the generation of high‐quality clinical evidence. Well‐designed, adequately powered randomized controlled trials are needed to evaluate the clinical efficacy of CAPP across different dental applications. Standardization of treatment parameters, including plasma source, gas composition, exposure duration, and delivery systems, is essential to improve reproducibility and comparability across studies.

Further investigations should also explore the underlying biological mechanisms in clinically relevant models, including molecular and transcriptomic analyses. In addition, long‐term safety assessments in human populations are required to establish the risk profile of repeated plasma exposure. Advances in device design and standardization may facilitate future clinical translation; however, such developments should be guided by robust clinical validation.

## 5. Conclusions

Preclinical evidence: In vitro and animal studies suggest that CAPP has antimicrobial, surface‐modifying, and potential regenerative effects in dental applications, with no significant safety concerns reported under experimental conditions.

Clinical evidence: Human data remains limited to a single randomized trial demonstrating short‐term periodontal benefits. As such, current evidence is insufficient to support routine clinical use. Further well‐designed clinical studies with standardized protocols are required to establish efficacy, safety, and long‐term outcomes.

Overall, while the risk‐of‐bias profile of included studies was generally low, the evidence base is predominantly preclinical, and conclusions regarding clinical applicability should be interpreted with caution.

## Funding

The authors declare that no financial support was received for the research and/or publication of this article. This work did not receive any specific grant from funding agencies in the public, commercial, or not‐for‐profit sectors.

## Conflicts of Interest

The authors declare no conflicts of interest.

## Data Availability

The original contributions presented in the study are included in the article. Further inquiries can be directed to the corresponding author.
